# Thrombocytosis as a Marker for Postoperative Complications in Colorectal Surgery

**DOI:** 10.1155/2018/1978639

**Published:** 2018-08-26

**Authors:** M. Mohamud, L. Osborne, H. G. Jones, A. Ahmed, J. Beynon, D. A. Harris, M. Evans, M. Davies, U. Khot, T. V. Chandrasekaran

**Affiliations:** Department of Surgery, Morriston Hospital, Heol Maes Eglwys, Morriston, Swansea SA6 6NL, UK

## Abstract

**Background:**

Blood platelet measurement is a widely available and inexpensive test that is performed routinely. Platelets are thought to act by inducing inflammation and play a role in clotting and antimicrobial defence. A postoperative rise in the platelet count (thrombocytosis) is often dismissed as an incidental finding, but there is growing evidence to suggest that it may act as an indicator to underlying pathology. It correlates with significant pyogenic infections as well as multiple malignancies. In addition to this, recent research indicates that thrombocytosis may be a useful prognostic indicator for postoperative outcomes in patients with malignancies. In patients undergoing surgery for gastric cancer, a combination of platelet count and neutrophil-to-lymphocyte (NLR) ratio collected preoperatively was shown to correlate with postoperative survival.

**Objective:**

To evaluate whether there is a positive correlation between pre- and postoperative thrombocytosis and the risk of complications following colorectal surgery.

**Methods:**

This was a retrospective observational study based in Morriston Hospital, Swansea. Patients undergoing elective colorectal surgery for an 18-month period between 2014 and 2016 were included. Data on patient demographics, pre- and postoperative platelet count, the first date at which the highest platelet count was recorded, length of stay, type of operation, and postoperative complications using the Clavien-Dindo classification was obtained from the theatre booking software (TOMS) and Welsh Clinical Portal. Pearson's chi-square test was used for the analysis of the categorical variables.

**Results:**

Of the 201 patients studied, 75 (37%) had postoperative thrombocytosis (platelets ≥ 500 × 10^9^/L, range 501–1136), 120 (59%) had postoperative normocytosis (platelets < 500 × 10^9^/L, range 107–499), and 6 (2.9%) patients were excluded due to insufficient data. Peak platelet level was seen at a median of 8 days postoperatively but ranged from days 1 to 49. In patients with thrombocytosis, the mean time to peak platelet count was 9.5 days and ranged 1 to 49 days. 101/195 (52%) patients had a Clavien-Dindo III/V postoperative complication: 63% patients with postoperative normocytosis and 24% with postoperative thrombocytosis. In the thrombocytosis group, 16/75 (21%) were found to have postoperative pelvic collections compared to 1/120 (0.8%) of the normocytic patients. The total percentage of medical complications (44% versus 20%, *p* = 0.006) and surgical complications (64% versus 15.8%, *p* = 0.0001) was higher in the thrombocytosis group compared to the normocytosis group.

**Conclusion:**

In this retrospective study, thrombocytosis was shown to have a positive correlation with postoperative medical and surgical complications. An elevated platelet count in the postoperative period should alert the clinician to a developing complication. We recommend that further studies with a larger sample size would test the specific associations with individual complications.

## 1. Introduction

Platelet count is a routinely performed blood test both before and after major surgery as part of the full (or complete) blood count. The finding of an elevated platelet count has little significance attached in day to day practice with clinicians placing more emphasis on other inflammatory markers such as the total white blood cell count, neutrophil count, and C-reactive protein. Platelets are thought to act by inducing inflammation and in doing so play a role in the body's antimicrobial defence [1] and may be implicated in the postoperative inflammatory response.

There is growing evidence to suggest that an increased platelet count may act as an indicator to underlying pathology [2, 3]. Thrombocytosis (high platelet count > 500 × 10^9^/L) is correlated with significant pyogenic infections such as empyema and abscess formation as well as acting as a possible precursor to soft tissue infections [4]. In addition, thrombocytosis is commonly seen in malignancy [5] and its use as a marker for predicting prognosis in rectal cancer [6], gynaecological malignancies [7–10], renal cell carcinomas [11], breast cancer [12], oesophageal cancer [13], gastric cancer [14], and lung cancer [15] is well documented.

Several papers have shown that thrombocytosis is a useful prognostic indicator for postoperative outcomes in patients with malignancy. In patients undergoing surgery for gastric cancer, a combination of platelet count and neutrophil to lymphocyte ratio (NLR) collected preoperatively was shown to correlate with postoperative survival [16]. Another study looked at postoperative platelet increase and its correlation to outcome in patients undergoing urological surgeries including radical cystectomy, percutaneous nephrolithotripsy, and nephrectomy [17]. In this study, there was a significantly higher rate of postoperative complications in patients who developed thrombocytosis after surgery compared to those who did not. There is little previously published about the implication of thrombocytosis after colorectal surgery.

## 2. Aim

The aim of this study was to evaluate whether there is a positive correlation between thrombocytosis and the development of complications after colorectal surgery.

## 3. Method

The study was based in the colorectal department at Morriston Hospital, a large secondary care university teaching hospital in Swansea, UK. We identified all patients who underwent elective colorectal surgery for an 18-month period between 2014 and 2016 from the national cancer registry (CANISC). Demographic details and operations performed were retrieved from the theatre booking software (TOMS database) to include management of complications. The variables assessed and recorded were pre- and postoperative platelet count, the first date at which the highest platelet count was recorded postoperatively, length of stay, type of operation, and complications using the Clavien-Dindo classification. We collected the information from the Welsh Clinical Portal (test result software) and the Synapse radiology portal. Patient case records were used to retrieve missing data. All the information gathered was stored in a password-protected spreadsheet and accessed from hospital computers. For statistical associations between categories of platelet count and individual complications, the Pearson's chi-square test was used. Statistical significance was assumed at the 5% level.

## 4. Results

Of the 201 patients studied, 75 (37%) had postoperative thrombocytosis (platelets ≥ 500 × 10^9^/L, range 501–1136), 4 (1.9%) of which had platelet count ≥ 1000 × 10^9^/L, 120 (59%) had postoperative normocytosis (platelets < 500 × 10^9^/L, range 107–499), and 6 (2.9%) patients were excluded due to insufficient retrievable data. 191 of the 201 patients studied had normal platelet count preoperatively.

Peak platelet level was seen at a median of 8 days postoperatively but ranged from 1 to 49 days. In patients with thrombocytosis, the mean time to peak platelet count was 9.5 days and range 1 to 49 days.

Out of the 195 patients included into this study, 123 (63.1%) had complications postoperatively while 72 (36.9%) did not. In the normocytosis group, 76/120 (63.3%) had no recorded complications compared to 18/75 (24%) in patients with thrombocytosis.

Complications were categorised into medical complications, surgical complications, and death. Medical complications included atelectasis, pleural effusion, lower respiratory tract infection, consolidation, pulmonary embolism, and deep vein thrombosis. 54/195 (27.6%) of patients in the study group had medical complications. However, 21/120 (17.5%) of the normocytosis group had a medical complication compared to 33/75 (44%) of patients who had thrombocytosis. Of the 44% of patients with thrombocytosis who had medical complications, 12% of these had consolidation. However, only 2.5% of patients with normocytosis had consolidation in their postoperative course as shown in Table 1.

Surgical complications categorised in this study were ileus, small bowel obstruction, collection, anastomotic leak, perforations, and hernia. Overall, 30.7% of patients studied had a surgical complication. This percentage was higher in patients with thrombocytosis at 56% and lower in the normocytosis group at 15%. Of patients with thrombocytosis, 21% had a postoperative collection compared to 0.85% of patients with normocytosis. As well as this, the percentage of anastamotic leaks (12%) and perforations (2.6%) was higher in patients in the thrombocytosis group compared to patients in the normocytosis group where there were no recorded cases of perforations or anastomotic leaks.

The percentage of patients who died in each group was similar, with a mortality of 17.4% among all patients studied. This was slightly lower for the normocytosis group (16.6%) compared to the thrombocytosis group (18.6%).

When the Clavien-Dindo classification was used to classify complications, the normocytosis group was more likely to have grade I complications at 68% than the thrombocytosis group (30%) as shown in Table 2. However, patients with thrombocytosis were more likely to have grade II and grade III complications at 28% and 22.6%, respectively, compared to patients with normocytosis who had grade II (9.1%) and grade III (4.1%) complications.

101/195 (52%) patients had a Clavien-Dindo III/V postoperative complication; 63% of patients with postoperative normocytosis and 24% with postoperative thrombocytosis. In the thrombocytosis group, 16/75 (21%) were found to have postoperative pelvic collections compared to 1/120 (0.8%) of the normocytic patients. The total percentage of medical complications (44% versus 20%, *p* = 0.006) and surgical complications (64% versus 15.8%, *p* = 0.0001) was higher in the thrombocytosis group compared to the normocytosis group.

## 5. Discussion and Conclusion

In this novel study, thrombocytosis was shown to have a positive correlation with postoperative medical and surgical complications following colorectal cancer surgery.

In the literature, platelets are thought to be an acute-phase reactant which increase in response to systemic insults such as inflammation, infections, neoplasms, and bleeding conditions [18–20]. This is what is termed as reactive or secondary thrombocytosis, and it is a benign state of thrombocytosis. Primary thrombocytosis (also known as essential thrombocytosis) is an unregulated abnormal production of platelets originating in the bone marrow. In normal states, thrombopoeisis (platelet production) is regulated by the hormone thrombopoeitin secreted from the liver and kidneys [19–21].

The pathophysiology behind secondary thrombocytosis (reactive thrombocytosis) which we are concerned with in this study is thought to be as a result of increased levels of proinflammatory cytokines, such as interleukins (IL-1, IL-6, and IL-11) as seen in inflammatory, infective, and malignant states [22–25]. The increase in these proinflammatory substances is thought to be the cause for the increased risk of developing surgical complications. The surgical and surrounding sites will inevitably experience increased levels of oedema postoperatively due to increased vascular permeability and coupled with the proinflammatory substances that are thought to lead to tissue damage. The nutrient-rich oedema can also act as the perfect culture medium for microorganisms to thieve which lead to further tissue destruction [26].

There is growing evidence to suggest that thrombocytosis states may act as an indicator to underlying pathological states, for example, septic and thromboembolic events [17]. An elevated platelet count should not merely be dismissed as a random occurrence in the postoperative period but should alert the duty clinician to a developing complication. They should actively seek out the cause through thorough history taking and clinical examination, and if indicated consider an urgent CT scan of the abdomen and pelvis [22–25].

The platelet count is an inexpensive and routinely collected proinflammatory marker to monitor for inflammatory and infective complications after surgery. We recommend that further studies with a larger sample size would test the specific associations with individual complications and try to define an absolute platelet level associated with complications.

## Figures and Tables

**Table 1:** Breakdown of complications.

Patients with platelets ≥ 500 × 10^9^/L *N* = 75			Patients with platelets < 500 × 10^9^/L *N* = 120			*p* value
*Medical complications*	*n*	*%*	*Medical complications*	*n*	*%*	
Atelectasis	9	12.0	Atelectasis	8	6.6	0.25
Consolidation	9	12.0	Consolidation	3	2.5	0.01
Effusion	8	10.6	Effusion	4	3.3	0.06
PE	6	8.0	PE	3	2.5	0.09
LRTI	1	1.3	LRTI	3	2.5	1.00

*Surgical complications*	*n*	*%*	*Surgical complications*	*n*	*%*	
Perforation	2	2.6	Perforation	0	0.0	0.15
Leak	9	12.0	Leak	0	0.0	0.00001
Small bowel obstruction	1	1.3	Small bowel obstruction	2	1.6	1.00
Collection	16	21.3	Collection	1	0.8	0.0001
Ileus	2	2.6	Ileus	0	0.0	0.15
Abscess	1	1.3	Abscess	1	0.8	1.00
Hernia	5	6.6	Hernia	4	3.3	0.31

**Table 2:** Complication rate according to Clavien-Dindo classification.

Clavien-Dindo class	*n*	%
*Patients with platelets ≥ 500 × 10^9^/L*		
I	23	30.6
II	21	28.0
III	17	22.6
IV	1	1.3
V	14	18.6
*Patients with platelets < 500 × 10^9^/L*		
I	82	68.3
II	11	9.1
III	5	4.1
IV	1	0.8
V	20	16.6

No significant difference was found between platelet count and the development of a Clavien-Dindo IV/V complication (*p* = 0.44).

## Data Availability

The data used to support the findings of this study are available from the corresponding author upon request.
